# Photosensitization With Supramolecular Arrays for Enhanced Antimicrobial Photodynamic Treatments

**DOI:** 10.3389/fbioe.2021.655370

**Published:** 2021-07-07

**Authors:** Cecilia Vera, Fiorella Tulli, Claudio D. Borsarelli

**Affiliations:** Instituto de Bionanotecnología del NOA (INBIONATEC), CONICET – Universidad Nacional de Santiago del Estero (UNSE), Santiago del Estero, Argentina

**Keywords:** microbial infections, antimicrobial resistance, photosensitization, supramolecular photosensitizers, antimicrobial photodynamic therapy

## Abstract

Microbial infections represent a silent threat to health that has worsened in recent decades due to microbial resistance to multiple drugs, preventing the fight against infectious diseases. Therefore, the current postantibiotic era forces the search for new microbial control strategies. In this regard, antimicrobial photodynamic therapy (aPDT) using supramolecular arrays with photosensitizing capabilities showed successful emerging applications. This exciting field makes it possible to combine applied aspects of molecular photochemistry and supramolecular chemistry, together with the development of nano- and biomaterials for the design of multifunctional or “smart” supramolecular photosensitizers (SPS). This minireview aims to collect the concepts of the photosensitization process and supramolecular chemistry applied to the development of efficient applications of aPDT, with a brief discussion of the most recent literature in the field.

## Antimicrobial Resistance: A Current Threat to Health

The misuse of antibiotics has contributed to the prevalence of superbugs that survive conventional pharmacological treatments, even new generation ones, due to the development of antimicrobial resistance (AMR) (WHO, 2014). AMR mechanisms include the molecular modification of antibiotics, binding sites and/or targets, changes in cell permeability that limit drug absorption and/or increase efflux, and biofilm formation (Peterson and Kaur, 2018). Currently, AMR is the leading cause of nosocomial profusion infections, such as pneumonia, tuberculosis, and malaria (Haque et al., 2018), increasing worldwide morbidity and mortality as well as healthcare costs (Guest et al., 2020).

Biofilm ecosystems of various bacteria, fungi, green algae, and lichens are responsible for almost 80% of human infections as a consequence of the protective effect of an extracellular matrix of polymeric substances (EPS) self-produced by the communities of microorganisms (Hall and Mah, 2017). Furthermore, low quality of drinking water is another source of microbial contamination with high social and economic impact (Prüss-Üstün et al., 2008), where various bacteria and viruses cause diarrheal and intestinal diseases (Tarrass and Benjelloun, 2012).

Therefore, the search for new approaches to control infections in different settings without the development of AMR and low postcontamination is essential. Bacteriocins, essential oils, bacteriophages, antibodies, quorum sensing inhibitors, and nanotherapeutics have been tested as effective antimicrobials without the development of AMRs, many of which receive Generally Recognized as Safe (GRAS) status. However, these methods are used successfully in combination with available antibiotics (Vivas et al., 2019).

## Light-Induced Treatments as Tools Against AMR

Light-based methods such as the use of ultraviolet C (UVC, 200–280 nm) and photodynamic therapy (PDT) act as efficient anti-infective treatments without the involvement of antibiotics (Yin et al., 2013). Standard germicidal UVC lamps emit primarily at 254 nm, a wavelength strongly absorbed by RNA and DNA in microbial cells, leading to dimer formation between pyrimidine residues in nucleic acid chains that alter the cell replication and eventually lead to cell death. However, for the same reason, UVC is very dangerous for mammalian cells, causing skin cancer (Apalla et al., 2017) and cataracts (Oriowo et al., 2001). Therefore, UVC disinfection with germicidal lamps is safe only for surfaces, although the new 222-nm monochromatic light sources showed excellent germicidal performance for bacteria and viruses without damaging effects on host cells (Buonanno et al., 2017).

**FIGURE 1:**
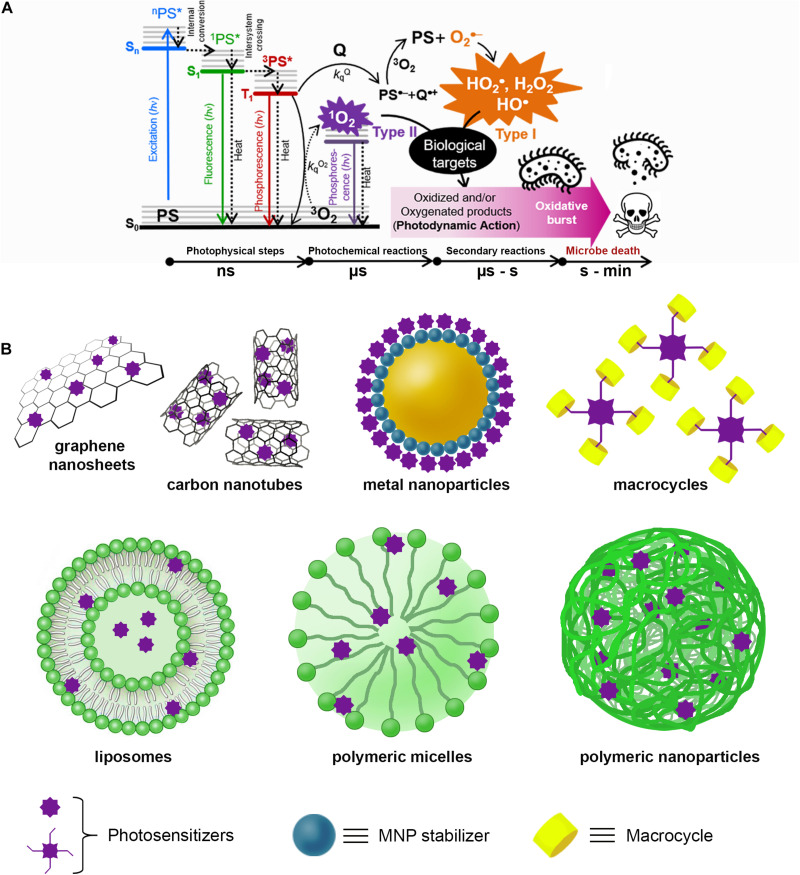
**(A)** Schematic representation of the antimicrobial photodynamic therapy (aPDT) process that begins with the absorption of a photon (*hν*) of appropriate wavelength by the ground state (S_0_) of the PS to populate any excited singlet state of higher energy (S_*n*_). Excess vibronic energy is released rapidly (< 1 ns) as heat by non-radiative processes to populate the lower singlet excitation state S_1_ (^1^PS*). Subsequently, the ^1^PS* state can decay to S_0_ on the nanosecond time scale by radiative (fluorescence) and non-radiative (heat processes), in parallel with the population of the excited state lower triplet T_1_ (^3^PS*) by an intersystem crossing process. In aerobic conditions, the reaction of ^3^PS* with a quencher molecule (Q) or molecular oxygen (^3^O_2_) by charge transfer (Type I) or energy-transfer (Type II) will produce reactive oxygen species (ROS), which in turn react with biological targets (lipids, DNA, proteins) inducing malfunctioning and, eventually, cell death. **(B)** Representative supramolecular architectures used for aPDT with PS non-covalently conjugated to carbon-based nanomaterials [e.g., carbon nanotubes (CNTs) and graphene sheets]; metal nanoparticles (e.g., gold, silver, copper, or platinum) stabilized with different compounds (e.g., amino acids, proteins, polymers, biopolymers, etc.); macrocycles (e.g., cyclodextrins, calixarenes, cucurbituriles, pillararenes, etc.); and finally, PS entrapped in liposomes, spherical micelles, and polymeric nanoparticles.

In contrast, the illumination of cells with wavelengths >400 nm is harmless, except in the presence of a photosensitizer molecule (PS) that absorbs light to produce photophysical or photochemical alterations in another molecular entity without any chemical modification of itself. This process is called photosensitization, and the key transient species is the lower triplet excited state T_1_ (^3^PS^∗^) of PS. In a biological medium, this relatively short-lived species (from hundreds of nano- to microseconds) can react with a biomolecule (Q) or molecular oxygen (^3^O_2_) either by Type I (charge-transfer) or Type II (energy-transfer) mechanisms, respectively, yielding reactive oxygen species (ROS), e.g., hydroxyl radical HO^⋅^, hydrogen peroxide H_2_O_2_, anion superoxide O_2_^⋅–^, and singlet oxygen ^1^O_2_ (Baptista et al., 2017; Figure 1A).

This photo-triggered ROS imbalance is responsible for the oxidation and/or oxygenation of biomolecules (DNA, lipids, proteins, etc.), producing cell malfunction and eventually cell death (Soriano et al., 2017). ROS-mediated degradation of biological substrates produced only by light excitation of a PS under aerobic conditions is termed photodynamic action (PDA), while the cell destruction produced by this effect is named photodynamic therapy (PDT) (Benov, 2015).

## Scope of Antimicrobial Photodynamic Therapy

Antimicrobial PDT (aPDT) refers to the killing of microbes mediated by PDA (Figure 1A). Although Oscar Raab’s first report on the use of light combined with acridine staining as a fast and efficient method to kill protozoa was in 1900, the development of PDT was overshadowed by the progress of clinical PDT for cancer (Cieplik et al., 2018). However, clinical interest in aPDT was recently renewed due to the AMR crisis (Kharkwal et al., 2011; Hu X. et al., 2018).

Due to the high reactivity of ROS and molecular crowding in the biological environment, photosensitized damage can be expected to occur near the location of PS (Awad et al., 2016). The molecular structure of PS and the composition of the outer wall of microbes are crucial for modulating the interaction of PS with the microbe, its intracellular compartmentalization and the degree of photodamage produced (Liu Y. et al., 2015; Kashef et al., 2017). Cationic PSs can photo-inactivate both Gram(+) bacteria and yeasts given the negative zeta potential values of their outer membranes (Dai et al., 2012; Cieplik et al., 2018). For Gram(−) bacteria, the extra dense negatively charged lipopolysaccharide outer membrane only allows the passage of small neutral hydrophilic molecules (Jori et al., 2011). In the case of biofilms, the EPS matrix also elicits molecular and electrostatic barriers to the PS penetration (Shrestha and Kishen, 2014; Supplementary Figure 1).

Depending on the localization of PS in the microbe, the photodamage can affect several critical targets. A PS embedded in the double-strand DNA can oxidize it by HO^⋅^ (Type I) and ^1^O_2_ (Type II) mediated reactions (Awad et al., 2016). Proteins are the most abundant biomolecules, and photoinduced ^1^O_2_-mediated injury depletes the enzymatic apparatus involved in the fermentative and glycolysis pathways, along with the loss of membrane barrier functions, compromising energy production and cell defense mechanisms (Dosselli et al., 2012). Photosensitized Type II oxidation modifies Trp, Tyr, Cys, His, and Met residues, resulting in loss of enzymatic activity, protein fragmentation and/or aggregation, changes in redox homeostasis, signaling, and cellular proteolytic pathways (Pattison et al., 2012). Photogenerated ROS also reacts with unsaturated membrane lipids to produce lipid hydroperoxides that can transform into shortened chain by-products that promote hydrophilic pore formation and/or membrane rupture (Itri et al., 2014; Di Mascio et al., 2019).

Therefore, efficient aPDT can be achieved by ROS-mediated non-specific damage to multiple cell targets with almost no possibility of AMR development. Other advantages of aPDT compared to conventional anti-infective treatments are the following: (i) a rapid (seconds to minutes) and spatially controlled light-activated microbial inactivation, (ii) a broader spectrum of action since the same PS can be active against several types of microorganisms, and (iii) efficient microbial depletion of >3log CFU, regardless of the antibiotic resistance patterns of the microbes (Hamblin and Abrahamse, 2019).

However, other factors can decrease the effectiveness of aPDT: (i) low chemical or photochemical stability of PS; (ii) low aqueous solubility of PS causing self-aggregation; (iii) low absorption of light by PS due to low extinction coefficient, light scattering or reflectance, internal filtering, etc.; (iv) low uptake of PS by microbes; and (v) hypoxic conditions at the PS location, a critical problem within biofilms where oxygen concentration can drop to 0.010 mM (de Beer et al., 1994), although cell photo-death under hypoxic conditions decreased Type I more than Type II reaction (Price et al., 2013).

## Supramolecular Chemistry Improves aPDT

Hundreds of organic and inorganic molecules are efficient PS, principally as ^1^O_2_ generators (Supplementary Figure 2 and Supplementary Table 1), e.g., tetrapyrroles; organic dyes; metal coordination complexes, quinones, isoalloxazines, BODIPYs dyes, phenalenones, and fullerenes, etc. (Redmond and Gamlin, 1999); although Type I oxidation processes are also relevant in the modification of nucleic acids (Serrano et al., 2015; Baptista et al., 2017) and proteins (Hawkins and Davies, 2001; Alarcón et al., 2009).

Owing to the complexity of biological media, it is unlikely that a single molecule of PS could fulfill all the characteristics required for an “ideal” application of aPDT. In this sense regard, the design of supramolecular photosensitizers (SPS) has provided adequate improvements in aPDT (Liu et al., 2013; Gao et al., 2019; Yao et al., 2019).

The concept behind supramolecular chemistry is to obtain reversible self- or coassembled functional structures through non-covalent interactions between more than two molecules and/or macromolecules. Generally, SPS can be formed by assembling a PS molecule with other molecular pieces through intermolecular forces that vary roughly in energy as follows: van der Waals < π–π stacking < dipole–dipole < π-cation < hydrogen bond < ion–dipole < ion–ion, in many cases acting cooperatively and reducing free energy to obtain the most thermodynamically stable aggregate (Supplementary Figure 3). Different supramolecular architectures can be obtained with size control from a few nano- to micrometer, e.g., 0D (nanoparticles), 1D (fibers), 2D (films, plates), and 3D (amorphous or defined-shaped composites). Due to the relative weakness of intermolecular interactions, supramolecules are reversibly formed through equilibria prone to respond to external stimuli, e.g., pH, temperature, ultrasound, redox agents, enzymes, etc., allowing extra control over the functionalization of the supramolecule (Albrecht, 2007; Li et al., 2018). Therefore, the functional control of an SPS by external stimuli offers the possibility to modulate the generation of ROS as suitable for each aPDT application (Li et al., 2018), besides producing synergistic antimicrobial effects by the supramolecular combination of PS with other inherently antimicrobial materials (antibiotics, nanoparticles, peptides, etc.) (Liu et al., 2013; Gao et al., 2019).

## Selected Examples of SPS Applied to aPDT

Figure 1B schematizes some typical SPS architectures, and Table 1 and Supplementary Table 2 summarize information of some representative SPS systems used in aPDT applications.

**TABLE 1:** Summary of properties of selected examples of supramolecular photosensitizers (SPS) used in antimicrobial photodynamic therapy (aPDT).

Photosensitizer (PS)	Supramolecular template	Type of interaction	Microorganism/antimicrobial efficiency	References
***Polymer-based materials***				
Cationic Zinc phthalocyanines	Cellulose nanocrystals	Electrostatic interactions	*S. aureus*: 6-3 logs *E. coli*: 8-6 logs *C. albicans*: 6.5 logs	Anaya-Plaza et al. (2017)
Ru(II) polypyridyl complexes	Porous silicone matrix	Hydrophobic interactions	*E. faecalis* Total disinfection	Manjón et al. (2014)
*meso-*tetraaryl porphyrins	Chitosan film	Electrostatic and H-bonding	*Listeria innocua* ≈ 2.5 logs Irradiation during attachment: 2-3 logs Irradiation after attachment: 1.5-2 logs	Castro et al. (2017)
***Self-assembled nanocarriers***				
Chlorophyll derivatives	Zwitterionic DPPC liposomes Non-ionic polymeric micelles	PS encapsulation	*S. aureus* ≈ 3 logs	Gerola et al. (2019)
			*S. aureus* ≈ 2 logs	
Aluminum Chloride Phthalocyanine	Tri-block copolymers (polymeric micelles)	PS encapsulation	*S. aureus* ≈ 3 logs *E. coli*: no effect *C. albicans*: <1 logs	Vilsinski et al. (2015)
Chlorin e6	Poly(HDDA-*co-*DBPA – mPEG) (polymeric nanoparticles)	PS encapsulation	*S. aureus* and *E. coli* Complete inhibition *in vivo:* Improved photodynamic therapeutic efficacy of NPs compared to free Ce6	Liu Y. et al. (2015)
***Macrocycles-based host-guest complexes***				
Cationic porphyrin derivative	Block polymer Backbone + cucurbit[8]uril	Host-guest complexation Metal coordination	*S. aureus* ≈ 100%	Chen et al. (2019)
Methylene blue	β-cyclodextrin-modified hyaluronic acid	Host-guest complexation	MRSA ≈ 2 logs	Yao et al. (2019)
***Carbon-based materials***				
Indocyanine green	Nano-Graphene Oxide	π-π stacking hydrophobic interactions	*E. faecalis* 2.81 logs 99.4%	Akbari et al. (2017)
***Metallic nanoparticles***				
Toluidine blue O	Silver nanoparticles	Electrostatic interactions	*S. mutans* 4 logs 99%	Misba et al. (2016)
Methylene blue	Gold nanoparticles	Electrostatic interactions	MRSA >5 logs reduction of MRSA 4-days-old biofilm	Darabpour et al. (2017)

*DBPA: 3-(Diethylamino)-1-propylamine; DPPC: dipalmitoylphosphatidylcholine; C. albicans: Candida albicans; E. coli: Escherichia coli; E. faecalis: Enterococcus faecalis; HDDA: 1,6-hexanediol diacrylate; L. innocua: Listeria innocua; MRSA: methicillin-resistant S. aureus; mPEG: methyl poly(ethylene glycol); S. aureus: Staphylococcus aureus.*

Among supramolecular self-assemblies, block copolymeric micelles made of pluronic surfactants, poly(ethyleneglycol) (PEG)–lipid conjugates, or pH-sensitive poly (N-isopropyl acrylamide) are emerging supramolecular systems highly attractive for physical entrapment and transport of PS (Van Nostrum, 2004) and nanomedicine applications (Cabral et al., 2018).

Aluminum phthalocyanine chloride (AlPcCl) was incorporated into polymeric micelles made of pluronic surfactants (P-123 and F-127), and the aPDT efficacy of these SPS against *Staphylococcus aureus*, *Escherichia coli*, and *Candida albicans* was investigated (Vilsinski et al., 2015). In the F-127 micelles, AlPcCl was self-aggregated and did not show an aPDT effect. In contrast, P-123 micelles can trap monomeric AlPcCl, and the formulation was effective against *S. aureus* and *C. albicans*.

The aPDT effect against *S. aureus* of chlorophyll derivatives (Chl) loaded in polymeric micelles of F-127 and dipalmitoylphosphatidylcholine (DPPC) liposomes was compared. The efficacy of both SPS depended on the structure of the Chl and its location in the supramolecular carrier, but the photodynamic activity was higher in liposomes bearing Chls without the phytyl chain (Gerola et al., 2019).

Although liposomes are more difficult and expensive to prepare than polymeric micelles (Kashef et al., 2017), they have several advantageous properties for use in aPDT, such as a high loading capacity of both hydrophobic and hydrophilic PS in the bilayer membrane or the aqueous core, respectively, biocompatibility, biodegradability, and suitable nanometric size (Jin and Zheng, 2011).

Molecular recognition based on “host–guest” interactions given by macrocyclic molecules was relevant to understanding supramolecular chemistry (Pedersen, 1967). In such systems, the macrocycle cavity size of <1 nm allows the intermolecular inclusion of a variety of PS as “guest” molecules using cyclodextrins, calix[*n*]arenes, pillar[*n*]arenes, [*n*]rotaxanes, and cucurbit[*n*]urils, leading to a variety of SPS formulations that normally increase Φ_Δ_ by preventing self-aggregation of PS and improving its solubility and photostability (Liu et al., 2013; Sowa and Voskuhl, 2020). Other notable characteristics of macrocycle-based SPS are that undesirable dark toxicity due to ionic substituent groups of PS can be reduced by the formation of the inclusion complex (Chen et al., 2017; Özkan et al., 2019). Moreover, on–off photosensitization control is possible by the convenient combination of PS–guest molecules and the internal diameter of the macrocycle (Robinson-Duggon et al., 2017).

Metal nanoparticles (MNP) not only can transport and release non-covalently attached PS but can also act as intrinsic antimicrobial agents due to the small size that allows them to adhere to the microbial wall, producing cell death through various mechanisms that can include the generation of ROS, disruption of transmembrane electron transport, the release of toxic species, and membrane modification resulting in a synergistic antimicrobial effect (Hajipour et al., 2012). The photodynamic effect of methylene blue–gold nanoparticles (AgNP@MB) on *S. aureus* isolated from impetigo lesions in children was evaluated using a diode laser at 660 nm as the excitation source (Tawfik et al., 2015). The “naked” AuNPs did not exert a direct bactericidal effect but improved the photodynamic activity of the MB adsorbed on the nanoparticle, possibly due to a photothermal effect due to the simultaneous excitation of the AuNPs.

Moreover, methicillin-resistant *S. aureus* (MRSA) in mature biofilms was efficiently eliminated (>5log CFU reduction) with the cationic dye MB immobilized on AgNP@citrate through electrostatic interactions. Results indicated enhanced ROS formation by the effect of localized surface plasmon resonance (SPR) of AuNPs (Darabpour et al., 2017). On the other hand, the antibiofilm action on *Streptococcus mutans* of a toluidine blue O-silver nanoparticle (AgNP@TBO) conjugate occurred via Type I mechanism through the generation of HO^⋅^ (Misba et al., 2016).

Carbon-based nanomaterials such as single- and multiwalled carbon nanotubes (SWCNT and MWCNT, respectively), graphene, fullerenes, etc. are also useful nanomaterials for SPS preparation due to their unique physicochemical, photochemical, mechanical, bacterial affinity, and electrical properties. For example, nanocomposites of TBO and MWCNT (MWCNT@TBO) were successfully used in aPDT for the eradication of both the planktonic cells and biofilms of *P. aeruginosa* and *S. aureus* (Anju et al., 2019). Indocyanine green (ICG) was incorporated into nano-graphene oxide (NGO), and effective aPDT against *Enterococcus faecalis* was obtained with very low ICG concentration, suggesting that the photodynamic action was enhanced in the nanocomposite (Akbari et al., 2017).

Finally, recent advances in the design and manufacture of nanomaterials and nanoobjects that facilitated the assembly of various types of SPS with enhanced aPDT potential have been reviewed (Lucky et al., 2015; Fakayode et al., 2018; Hu B. et al., 2018; Mesquita et al., 2018).

## *In vivo* and Clinical aPDT Applications With SPS

The aPDT effectiveness of many PS has been tested *in vivo* using infected mice as animal models (Huang et al., 2010), and cationic liposome preparations have been used as selective delivery systems that favor interaction with negatively charged microbial cells rather than eukaryotic cells, optimizing PS uptake by bacteria and allowing its use for *in vivo* aPDT applications even for Gram(−) bacteria (Vimaladevi et al., 2016). Furthermore, the aPDT effect of hypericin-loaded amphiphilic block copolymer nanoparticles was tested *in vivo* in MRSA-infected wounds in rats, showing faster healing, better epithelialization, keratinization, and collagen fiber development (Nafee et al., 2013). A dual-mode antibacterial effect was tested for a nanocomposite made with a cationic porphyrin bound to graphene nanoribbons against *A. baumannii* and MRSA by the synergy of PDA with photothermal effects (Yu et al., 2020).

Induction of bladder infection was performed in mice to model acute cystitis as a typical case of urinary tract infection to evaluate the performance of Chlorine e6 encapsulated in polymeric nanoparticles, and a significant drop in bacterial cells was observed with minimal side effects (Liu S. et al., 2015).

The application of aPDT in dentistry has been extensively reviewed (Carrera et al., 2016), and it was concluded that topical aPDT has several advantages such as versatility, low cost, and the absence of harmful effects for patients. Various SPS systems composed of organic dyes as PS in self-assembled nanocarriers (micelles, liposomes, etc.) were used, such as AlClPc entrapped in cationic liposomes, which was effective in treating various oral infections (Longo et al., 2012).

Examples of clinical applications of aPDT for the control of various infections can be found in various reviews by Hamblin and collaborators (Kharkwal et al., 2011; Dai et al., 2012; Yin and Hamblin, 2015; Hu X. et al., 2018; Hamblin and Abrahamse, 2019). Briefly, it must be considered that nosocomial infections generated by contaminated surfaces are of great concern, since pathogens can survive for a long time on surgical surfaces and instruments and their adequate disinfection is essential (Haque et al., 2018). Regarding this, excellent aPDT activity has been tested for decontamination of *S. aureus* on a surface by spray coating made of polymeric micelles of PEG monomethyl ether-co-polylactide branched copolymers loading BODIPYs even at micromolar concentrations (Cabral et al., 2018). Also for surface disinfection, macrocycles have been used as supramolecular linkers to give rise to larger functional materials for aPDT with spray coatings (Yao et al., 2019) and fabric fibers (Castriciano et al., 2017). The inactivation and removal of biofilms in surgical elements are of great concern, as these microbe communities are particularly difficult to kill with antibiotics (Stewart and Costerton, 2001). The microbial cells within the biofilm are firmly attached to the substrate and embedded in a protective barrier made of an extracellular polymeric matrix of polysaccharides, proteins, and extracellular DNA (Rabin et al., 2015). Despite this, it has been shown that ROS generated in aPDT can attack the biofilm matrix, cell surface, and cytoplasm, damaging non-specific targets that lead to degradation of both planktonic cells and biofilms (Hu X. et al., 2018; Gao et al., 2019).

## Summary and Outlook

Antimicrobial PDT using SPS, many of them based on nanometric scaffolds of a wide variety of materials, emerges as a rapid and spatially precise methodology for the control of a wide spectrum of infections in different environments (Lucky et al., 2015; Fakayode et al., 2018; Hu B. et al., 2018; Mesquita et al., 2018). According to the Scopus^®^ database, research interest in “supramolecular photosensitization” has been increasing over the past two decades (Supplementary Figure 4), driven by the need for effective and less invasive anticancer and antimicrobial treatments.

The continuous development of new synthetic and biocompatible nanomaterials allows the design of “smart” SPS with multiple functionalities that respond to external stimuli other than light (e.g., pH, temperature, ionic strength, etc.) that can improve efficiency of aPDT by the controlled tuning of surface charges and adhesion to microbes, PS transport, and controlled delivery, improvement of photosensitizing properties, and synergistic antimicrobial effects in addition to PDA, such as intrinsic dark toxicity of the composite, photothermal effect, etc. (Liu et al., 2013; Abrahamse and Hamblin, 2016; Feng et al., 2017; Hu B. et al., 2018).

Finally, due to the multitarget oxidative action of photogenerated ROS, it is believed that aPDT does not develop AMR (Cieplik et al., 2018), but in any case, the question is how long aPDT can be applied without induction of survival mechanisms (Hamblin and Abrahamse, 2019). Therefore, given the current microbial crisis, the continued renewal of the aPDT requires interdisciplinary efforts involving chemists, microbiologists, and physicians, among others.

## Author Contributions

CV and FT contributed to figures and manuscript draft preparations. CDB wrote and revised the manuscript. All authors approved it for publication.

## Conflict of Interest

The authors declare that the research was conducted in the absence of any commercial or financial relationships that could be construed as a potential conflict of interest.
