# Supplementary material for: Photosensitization With Supramolecular Arrays for Enhanced Antimicrobial Photodynamic Treatments
Source: Front Bioeng Biotechnol. 2021 Jul 7;9:655370. doi: 10.3389/fbioe.2021.655370 (PMC8293899; doi:10.3389/fbioe.2021.655370)
Supplement: Supplementary file 1 [file Data_Sheet_1.docx]

Supplementary material

Photosensitization With Supramolecular Arrays for Enhanced Antimicrobial Photodynamic Treatments

Cecilia Vera,^†^ Fiorella Tulli, and Claudio D. Borsarelli*

Instituto de Bionanotecnología del NOA (INBIONATEC). CONICET - Universidad Nacional de Santiago del Estero (UNSE); Argentina.

***Correspondence:**Corresponding Author: Claudio D. Borsarelli
[cdborsarelli@gmail.com](mailto:cdborsarelli@gmail.com)

^†^These authors have contributed equally to this work

**Supplementary Figures**


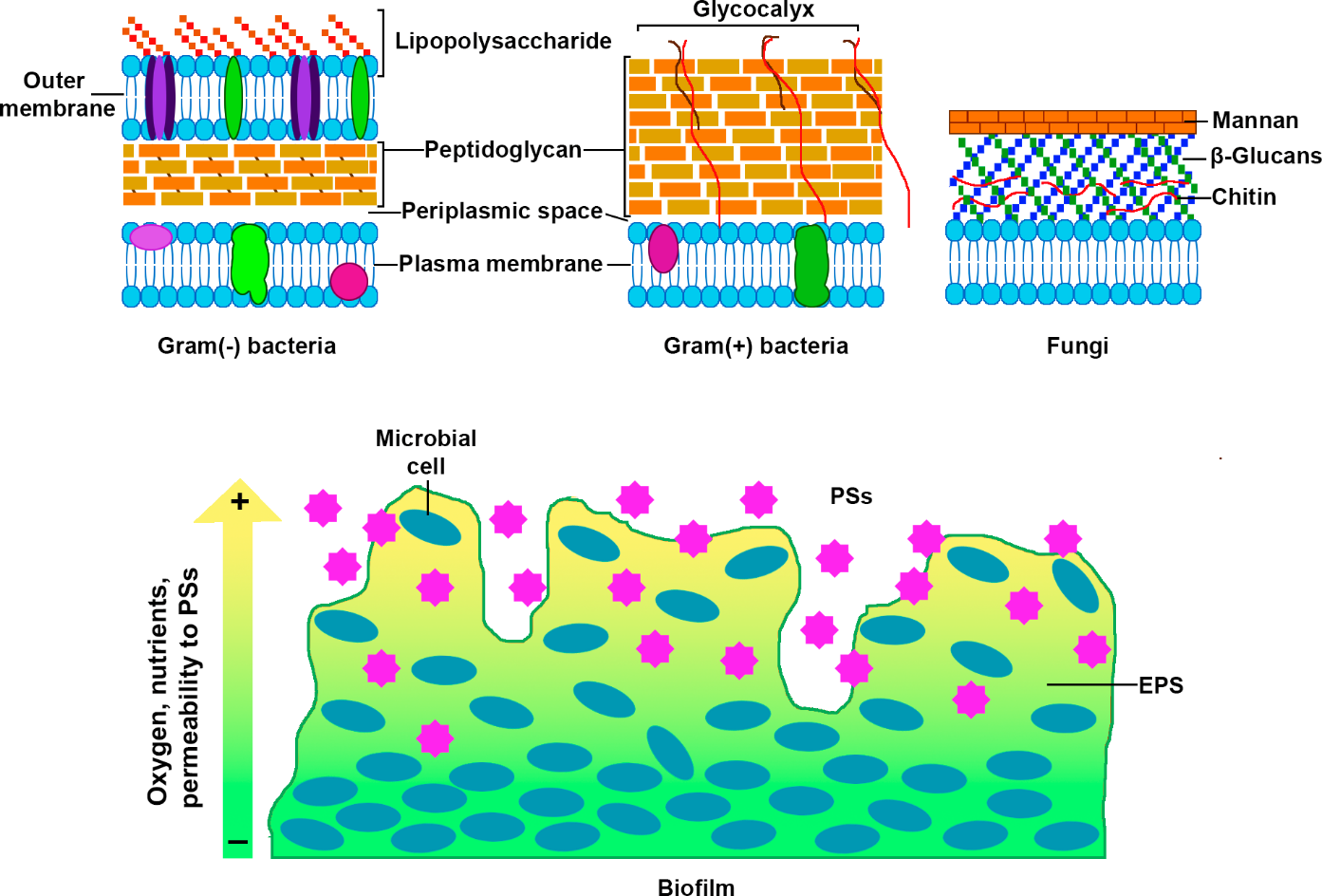


**Figure S1:** Schematic representation of the cell wall of Gram (+) and Gram (-) bacteria, fungi, and bacterial biofilm indicating the biopolymeric barriers for PS internalization in each case.

**

**

**Figure S2:** Molecular structure, absorption spectra, and singlet oxygen quantum yield (Φ_Δ_) of representative organic photosensitizers (PS) with light-absorbing properties in the visible region


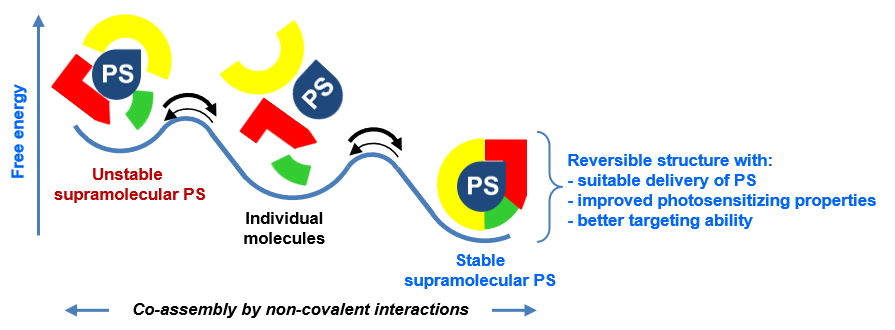


**Figure S3:** Schematic representation of the thermodynamically controlled formation of a supramolecular photosensitizer (SPS), where individual components, such as puzzle pieces, will fit together if their shapes are complementary (molecular recognition), while the best fit of the pieces will be obtained from the right way if the process is dynamically reversible (non-covalent interactions) until the most energetically stable SPS is obtained, producing the greatest decrease in free energy Δ*G* (co-assembly). Adapted from (Albrecht, 2007).





**Figure S4:** Number of articles published since 1985 in Scopus database obtained by searching of the terms “molecular photosensitization” and ‘‘supramolecular photosensitization”, respectively.

**Supplementary Tables**

**Table S1.** Basic chemical structure, solvent, excitation wavelength, and singlet oxygen quantum yields of selected photosensitizers

| **PS structure** | **Photosensitizer** | **Solvent** | **λ_ex_ (nm)** | **Φ_Δ_^(Ref)^** |
| --- | --- | --- | --- | --- |
| 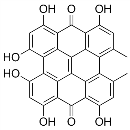Perylenequinones | Hypericin  Hypericin  Hypericin  Di-cupper hypericin  Calphostin C | Brij35 micelles  EtOH  DMF  EtOH  EtOH | 308  308  550  436 | 0.72^(a)^  0.73^(b)^  0.23^(a)^  0.90^(a)^  0.74^(a)^ |
| 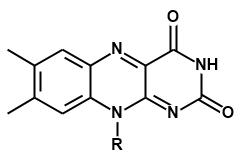Flavins | Lumiflavin  6-methylalloxazine  Riboflavin  Flavin monocleotide | EtOH  CH_3_CN  PBS pH 7.4  PBS pH 7.4 | 436  355  445  445 | 0.80^(a)^ 0.78^(a)^  0.49^(a)^  0.49^(a)^ |
| 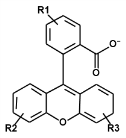Xanthenes | Fluorescein  Erythrosin  Eosin Y  Rose Bengal  4,5-diBr-rhodamine methyl ester | MeOH  H_2_O  H_2_O  PBS pH 7.4  MeOH | 504  500  540  514 | 0.09^(a)^  0.63^(a)^  0.50^(a)^  0.75^(a)^  0.47^(a)^ |
| 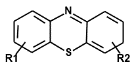Phenothiazines | Methylene Blue  Methylene Blue  Thionine  4-Iodomethylene Violet  Toluidine Blue | EtOH  PBS/TX100  H_2_O  EtOH  H_2_O | 577  630  580  630  660 | 0.50^(a)^  0.49^(b)^  0.58^(a)^  0.49^(a)^  0.60^(b)^ |
| 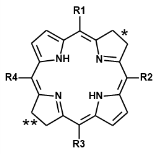Tetrapyrroles  Porphyrins  with C=C at * and **  Bacteriochlorins  without C=C at * and **  Chlorins  with C=C only at **  Phthalocyanines  with benzene ring at * and ** | Hematoporphyrin  Hematoporphyrin 8 μM  Hematoporphyrin 400 μM  Hematoporphyrin 30 μM  Photofrin®  Photofrin®  Photofrin® II  Photofrin® II  Tetrakis(4-sulfonatophenyl)porphyrin  Uroporphyrin IX  Bacteriochlorin  Chlorin *e*_6_  Chlorin *e*_6_ dimethyl ester  Tin (IV) chlorin *e*_6_  Zinc chlorin *e*_6_  Phthalocyanine  Phthalocyanine sulfonate  Phthalocyanine tetrasulfonate  Mg (II) phthalocyanine  Zn(II) sulfo-phthalocyanine  Zn(II) bis(pyridine) phthalocyanine  Zn(II) bis(pyridine) phthalocyanine | EtOH  H_2_O  H_2_O  EPC liposomes  PBS/TX100  Polystyrene microspheres  D_2_O  H_2_O/EPC liposomes  D_2_O  PBS pH 7.4  MeOD/air sat  EtOH  EtOH  PBS/D_2_O  D_2_O  MeOD  D_2_O  MeOD  C_5_H_5_N/O_2_  H_2_O pH 7  EtOH  DPPC vesicles | 632  576  576  546  630  514  632  532  532  355  532  532  355  532  347  670  347  669  670  600  600 | 0.57^(a)^  0.42^(a)^  0.19^(a)^  0.77^(a)^  0.25^(a)^  0.84^(a)^  0.16^(a)^  0.19^(a)^  0.64^(a)^  0.52^(a)^  0.62^(a)^  0.65^(a)^  0.70^(a)^  0.82^(a)^  0.82^(a)^  0.16^(a)^  0.14^(a)^  0.17^(a)^  0.40^(b)^  0.45^(b)^  0.53^(b)^  0.70^(b)^ |
| 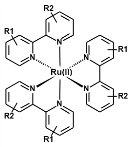  Ruthenium (II)-tris-bipyridyls | [tris(bipyridine)Ru(II)]Cl_2_  [tris(bipyridine)Ru(II)]Cl_2_  [tris(bipyridine)Ru(II)]Cl_2_  [tris(bipyridine)Ru(II)]Cl_2_ | CH_3_CN  H_2_O  CTAC micelles  SDS micelles | 450  355  450  450 | 0.57^(c)^  0.29^(d)^  0.30^(e)^  0.34^(e)^ |

References: ^(a)^ (Redmond and Gamlin, 1999); ^(b)^ (Wilkinson et al., 1993); ^(c)^ (Abdel-Shafi et al., 2001); ^(d)^ (Giménez et al., 2016); and ^(e)^ (Gutierrez et al., 2003)

**Table S2:** Summary of properties some of supramolecular photosensitizers (SPS) used in antimicrobial photodynamic therapy (aPDT)

| **Photosensitizer (PS)** | **Supramolecular template** | **Type of**  **interaction** | **Microorganism/**  **antimicrobial**  **efficiency** | **Experimental**  **conditions** | **Reference** |
| --- | --- | --- | --- | --- | --- |
| ***Supramolecular photosensitizers containing inorganic materials*** | | | | | |
| Toluidine blue O | Silver nanoparticles | Electrostatic | *S. mutans ≈*4 log^p^^[[1]](#footnote-1)^ 99%^ba^ | 360 nm laser light Output power: 100 mW/cm^2^  Energy dose: 9.1 J/cm^2^ | (Misba et al., 2016) |
| Methylene blue  Toluidine blue O | Gold nanoparticles | Electrostatic | *C. albicans* ≈0.5 log^p^, ≈80%^ba, mb^ *in vivo:* 50% reduction of CFU | Visible light source with filters: 662 nm for MB and 635 nm for TBO Output power: 120 mW Irradiance: 180 W/m^2^ Energy dose: 21.6 J/cm^2^ *in vivo* a mouse model | (Sherwani et al., 2015) |
| Methylene blue | Gold nanoparticles | Non-covalent | *S. aureus* from impetigo lesions 97%^p^ | 660 nm laser diode Output power: 200 mW Energy dose: 24 J/cm^2^ | (Tawfik et al., 2015) |
| Methylene blue | Gold nanoparticles | Electrostatic | MRSA >5 log^mb^ | 650 nm laser diode Output power: 30 mW Energy dose: 22.9 J/cm^2^ | (Darabpour et al., 2017) |
| Indocyanine green | Nano-Graphene Oxide | π-π stacking hydrophobic | *E. faecalis ≈*2.81 logs^p^ 99.4%^ba^ | 810 nm diode laser  Output power: 250 mW  Irradiated area: 038 cm^2^ Irradiation time: 60 s | (Akbari et al., 2017) |
| Toluidine blue | Multiwalled carbon nanotube | n.s. | *P. aeruginosa* ≈4.91 log^p^, 69.9%^ba^ *S. aureus* ≈5.5 log^p^ ,75.54%^ba^ | 670 nm laser, 125 mW  Irradiated area: 0,384 cm^2^ Irradiation time: 3 min | (Anju et al., 2019) |
| Indocyanine green | Carnosine-GO conjugates + hydroxyapatite | n.s. | *S. mutans* ≈95.5%^p^, 63.8%^ba^ | 810 nm Diode laser system Output power: 250 mW Energy dose: 31.2 J/cm^2^ | (Gholibegloo et al., 2018) |
| Methylene blue | Silicone | n.s. | *S. epidermidis* ≈44%^ba^ | 660 nm laser  Output power: 230 mW Total Energy dose: 117 J/cm^2^ | (Perni et al., 2010) |
| Ru(II) polypyridyl complexes | Porous silicone | Hydrophobic | *E. faecalis* Total disinfection | 150 W Xe lamp Irradiance: 20 W/m^2^ (360-700 nm), Irradiation time: 9 h | (Manjón et al., 2014) |
|  | | | | | |
| ***Supramolecular photosensitizers containing organic materials*** | | | | | |
| Cationic porphyrin derivative | Block Polymer Backbone + cucurbit[8]uril | Host-guest complexation Metal coordination | *S. aureus* ≈100%^mb^ | White light (halogen lamp) Irradiance: 20 mW/cm^2^ Irradiation time: 3 min | (Chen et al., 2019) |
| Methylene blue | β-cyclodextrin-modified hyaluronic acid | Host-guest complexation | MRSA ≈2 logs^p^ | 660 nm laser Energy dose: 30 J/cm^2^  Φ_∆_= 0.55 ± 0.02^[[2]](#footnote-2)^ | (Yao et al., 2019) |
| Cationic porphyrin derivative | Cucurbit[7]uril | Host-guest complexation | *E. coli* ≈97%^p^ | White light Irradiance: 25 mW/cm^2^ Energy dose: 1 J/cm^2^ (40 s) | (Liu et al., 2013) |
| Cationic porphyrin derivative | Captisol | Host-guest complexation | *E. coli* ≈81%^p^ | White LED, ≈50 mW/cm^2^ Irradiation time: 5 min  Φ_∆_= 0.95 (DMF) | (Khurana et al., 2019) |
| Hydrophilic and hydrophobic porphyrinoids | Amphiphilic calix[4]arene | Host-guest complexation | *P. aeruginosa* and  *S. aureus* 100%^p^ | 470 W Xenon lamp with a cut-off filter at 400 nm Irradiation time ≤30 min | (Di Bari et al., 2016) |
| Chlorin e6 | Polyethylene glycol bound to Magainin I | Host-guest complexation | *P. aeruginosa* 6.72 log^mb^ MRSA 6.59 log^mb^ | 660 nm laser Irradiation time: 8 min | (Gao et al., 2019) |
| Cationic porphyrin | Cucurbit[7]uril | Host-guest complexation | *E. coli* >90%^p^ | White light Irradiance: 40 mW/cm^2^ Irradiation time: 1 min | (Chen et al., 2017) |
| Anionic porphyrin (TPPS) | Polypropylene fabric with citrate-hydroxypropyl-βCD polymer | Host-guest complexation | *S. aureus* ≈99.98%^p^ | 50 W halogen lamp with a cut-off filter at 340 nm and water-filled 1 cm cell Energy dose: ≈5 J/cm2 Irradiation time: 30 min | (Castriciano et al., 2017) |
| Rhodamine 6G | Liposomes from hen egg-extracted lipids | Encapsulation | Multi-drug resistant *P. aeruginosa* OD_600_ drop from 0.9 to 0.1-0.3^p^ | 150 W Xe arc lamp in the visible region (KI + pyridine cut-off filters) Irradiance: 30 mW/cm^2^ Irradiation time: 10 min  Φ_∆_= 0.61 | (Vimaladevi et al., 2016) |
| Aluminum-chloride- phthalocyanine | Cationic liposomes | Encapsulation | Infected cariogenic dentine isolates≈50%^p^  Clinical: 82% average reduction of total cariogenic bacteria in dentin carious lesions | 660 nm continuous laser Energy dose: 20.6 J/cm^2^ Clinical: 10 human patients presenting class I cavitated carious lesions. Energy dose 180 J/cm^2^. | (Longo et al., 2012) |
| Chlorophyll derivatives | Zwitterionic DPPC liposomes | Encapsulation | *S. aureus* ≈3 log^p^ | Diode laser (659 nm, 100 mW) Energy dose: 153, 306, and 459 J/cm^2^ | (Gerola et al., 2019) |
| Aluminum Chloride Phthalocyanine | Tri-block copolymers | Hydrophobic with co-polymer sites | *S. aureus*  3 log 15 min^p^ *E. coli*: no effect *C. albicans*  <1 log^p^ | 659 nm diode laser Output power: 100 mW Irradiated area: 1.77 cm^2^ Irradiance: 70.7 mW/cm^2^ Irradiation time: 15 min | (Vilsinski et al., 2015) |
| Chlorophyll derivatives | Non-ionic polymers | Encapsulation | *S. aureus* ≈2 log^p^ | Diode laser (λ_max_=659 nm, 100 mW) Energy dose: 153, 306, and 459 J/cm^2^ | (Gerola et al., 2019) |
| Chlorin e6 | Poly(HDDA-*co-*DBPA—mPEG) | Encapsulation | *S. aureus* and *E. coli* Complete inhibition^p^ *in vivo:* Improved photodynamic therapeutic efficacy of NPs compared to free Ce6 | 660 nm laser light  Irradiance: 50 mW/cm^2^ Irradiation time: 10 min *in vivo:* Mouse acute cystitis model | (Liu et al., 2015) |
| Methylene blue | Poly(lactic-*co-*glycolic acid) | Encapsulation | Bacteria isolated from dental plaque 0.71 log^p^ 0.69 log^mb^ Clinical: improvement on clinical parameters after 1 month of treatment | 660 nm diode laser Irradiance: 100 mW/cm^2^ Energy dose: 20 J/cm^2^ Clinical: Human patients with moderate to advanced chronic periodontitis | (de Freitas et al., 2016) |
| Hypericin | Amphiphilic block co-polymers | Encapsulation | Clinical MRSA isolates <40%^p^ 20-100%^mb^ variable among isolates *in vivo:* Increased wound healing potential, better epithelialization, and keratinization of skin layers compared to free PS | Halogen lamp Output power: 20 W Irradiation time: variable *in vivo:* Infected wounds in female Winstar rats. Energy dose: 25.5 J/cm2. | (Nafee et al., 2013) |
| Cationic Zinc phthalocyanines | Cellulose nanocrystals | Electrostatic | *S. aureus:* 6-3 log^p^ *E. coli:* 8-6 log^p^ *C. albicans:* 6.5 log^p^ | Red light (620-645 nm) Irradiance: 18 mW/cm^2^ Irradiation time: 60 min | (Anaya-Plaza et al., 2017) |
| *meso-*tetraaryl porphyrins | Chitosan film | Electrostatic and H-bonding | *L. innocua* ≈2.5 log^p^ 2-3 log^ba^ , 1.5-2 log^mb^ | White LED (400-800 nm) Irradiance: 10 mW/cm^2^ Irradiation time: 24 h | (Castro et al., 2017) |

***Abbreviations:***

DBPA: 3-(Diethylamino)-1-propylamine;

DPPC: dipalmitoylphosphatidylcholine;

*C. albicans: Candida albicans;*

*E. coli: Escherichia coli;*

*E. faecalis: Enterococcus faecalis;*

HDDA: 1,6-hexanediol diacrylate;

*L. innocua: Listeria innocua;*

MRSA: methicillin-resistant *S. aureus*;

mPEG: methyl poly(ethylene glycol);

*P. aeruginosa: Pseudomona aeruginosa*

*S. aureus: Staphylococcus aureus,*

*S. epidermidis: Staphylococcus epidermidis;*

*S. mutans: Streptococcus mutans*

**References**

Abdel-Shafi, A.A., Beer, P.D., Mortimer, R.J., Wilkinson, F., 2001. Photosensitized generation of singlet oxygen from (substituted bipyridine)ruthenium(II) complexes. Helv. Chim. Acta 84, 2784–2795.

Akbari, T., Pourhajibagher, M., Hosseini, F., Chiniforush, N., Gholibegloo, E., Khoobi, M., Shahabi, S., Bahador, A., 2017. The effect of indocyanine green loaded on a novel nano-graphene oxide for high performance of photodynamic therapy against Enterococcus faecalis. Photodiagnosis Photodyn. Ther. 20, 148–153.

Albrecht, M., 2007. Supramolecular chemistry—general principles and selected examples from anion recognition and metallosupramolecular chemistry. Naturwissenschaften 94, 951–966.

Anaya-Plaza, E., van de Winckel, E., Mikkilä, J., Malho, J.M., Ikkala, O., Gulías, O., Bresolí-Obach, R., Agut, M., Nonell, S., Torres, T., Kostiainen, M.A., de la Escosura, A., 2017. Photoantimicrobial Biohybrids by Supramolecular Immobilization of Cationic Phthalocyanines onto Cellulose Nanocrystals. Chem. Eur. J. 23, 4320–4326.

Anju, V.T., Paramanantham, P., Sruthil, S.L., Sharan, A., Syed, A., Bahkali, N.A., Alsaedi, M.H., K., K., Busi, S., 2019. Antimicrobial photodynamic activity of toluidine blue-carbon nanotube conjugate against Pseudomonas aeruginosa and Staphylococcus aureus - Understanding the mechanism of action. Photodiagnosis Photodyn. Ther. 27, 305–316.

Castriciano, M.A., Zagami, R., Casaletto, M.P., Martel, B., Trapani, M., Romeo, A., Villari, V., Sciortino, M.T., Grasso, L., Guglielmino, S., Scolaro, L.M., Mazzaglia, A., 2017. Poly(carboxylic acid)-Cyclodextrin/Anionic Porphyrin Finished Fabrics as Photosensitizer Releasers for Antimicrobial Photodynamic Therapy. Biomacromolecules 18, 1134–1144.

Castro, K.A.D.F., Moura, N.M.M., Fernandes, A., Faustino, M.A.F., Simões, M.M.Q., Cavaleiro, J.A.S., Nakagaki, S., Almeida, A., Cunha, Â., Silvestre, A.J.D., Freire, C.S.R., Pinto, R.J.B., Neves, M. da G.P.M.S., 2017. Control of Listeria innocua biofilms by biocompatible photodynamic antifouling chitosan based materials. Dye. Pigment. 137, 265–276.

Chen, L., Bai, H., Xu, J.-F., Wang, S., Zhang, X., 2017. Supramolecular Porphyrin Photosensitizers: Controllable Disguise and Photoinduced Activation of Antibacterial Behavior. ACS Appl. Mater. Interfaces 9, 13950–13957.

Chen, L., Yang, Y., Zhang, P., Wang, S., Xu, J.F., Zhang, X., 2019. Degradable supramolecular photodynamic polymer materials for biofilm elimination. ACS Appl. Bio Mater. 2, 2920–2926.

Darabpour, E., Kashef, N., Amini, S.M., Kharrazi, S., Djavid, G.E., 2017. Fast and effective photodynamic inactivation of 4-day-old biofilm of methicillin-resistant Staphylococcus aureus using methylene blue-conjugated gold nanoparticles. J. Drug Deliv. Sci. Technol. 37, 134–140.

de Freitas, L.M., Calixto, G.M.F., Chorilli, M., Giusti, J.S.M., Bagnato, V.S., Soukos, N.S., Amiji, M.M., Fontana, C.R., 2016. Polymeric nanoparticle-based photodynamic therapy for chronic periodontitis in Vivo. Int. J. Mol. Sci. 17.

Di Bari, I., Fraix, A., Picciotto, R., Blanco, A.R., Petralia, S., Conoci, S., Granata, G., Consoli, G.M.L., Sortino, S., 2016. Supramolecular activation of the photodynamic properties of porphyrinoid photosensitizers by calix[4]arene nanoassemblies. RSC Adv. 6, 105573–105577.

Gao, Y., Wang, J., Hu, D., Deng, Y., Chen, T., Jin, Q., Ji, J., 2019. Bacteria-Targeted Supramolecular Photosensitizer Delivery Vehicles for Photodynamic Ablation Against Biofilms. Macromol. Rapid Commun. 40, 1800763.

Gerola, A.P., Costa, P.F.A., de Morais, F.A.P., Tsubone, T.M., Caleare, A.O., Nakamura, C. V., Brunaldi, K., Caetano, W., Kimura, E., Hioka, N., 2019. Liposome and polymeric micelle-based delivery systems for chlorophylls: Photodamage effects on Staphylococcus aureus. Colloids Surfaces B Biointerfaces 177, 487–495.

Gholibegloo, E., Karbasi, A., Pourhajibagher, M., Chiniforush, N., Ramazani, A., Akbari, T., Bahador, A., Khoobi, M., 2018. Carnosine-graphene oxide conjugates decorated with hydroxyapatite as promising nanocarrier for ICG loading with enhanced antibacterial effects in photodynamic therapy against Streptococcus mutans. J. Photochem. Photobiol. B Biol. 181, 14–22.

Giménez, R.E., Vargová, V., Rey, V., Turbay, M.B.E., Abatedaga, I., Morán Vieyra, F.E., Paz Zanini, V.I., Mecchia Ortiz, J.H., Katz, N.E., Ostatná, V., Borsarelli, C.D., 2016. Interaction of singlet oxygen with bovine serum albumin and the role of the protein nano-compartmentalization. Free Radic. Biol. Med. 94, 99–109.

Gutierrez, M., Martinez, C., Fresnadillo, D., Castro, A.M., Orellana, G., Braun, M., Oliveros, E., 2003. Singlet Oxygen (1∆g) Production by Ruthenium (II) Complexes in Microheterogeneous Systems. J.Phys. Chem 107, 3397–3403.

Khurana, R., Kakatkar, A.S., Chatterjee, S., Barooah, N., Kunwar, A., Bhasikuttan, A.C., Mohanty, J., 2019. Supramolecular nanorods of (N-methylpyridyl) porphyrin with captisol: Effective photosensitizer for anti-bacterial and anti-tumor activities. Front. Chem. 7, 452.

Liu, K., Liu, Y., Yao, Y., Yuan, H., Wang, S., Wang, Z., Zhang, X., 2013. Supramolecular photosensitizers with enhanced antibacterial efficiency. Angew. Chemie Int. Ed. 52, 8285–8289.

Liu, S., Qiao, S., Li, L., Qi, G., Lin, Y., Qiao, Z., Wang, H., Shao, C., 2015. Surface charge-conversion polymeric nanoparticles for photodynamic treatment of urinary tract bacterial infections. Nanotechnology 26, 495602.

Longo, J.P.F., Leal, S.C., Simioni, A.R., De Fátima Menezes Almeida-Santos, M., Tedesco, A.C., Azevedo, R.B., 2012. Photodynamic therapy disinfection of carious tissue mediated by aluminum-chloride-phthalocyanine entrapped in cationic liposomes: An in vitro and clinical study. Lasers Med. Sci. 27, 575–584.

Manjón, F., Santana-Magaña, M., García-Fresnadillo, D., Orellana, G., 2014. Are silicone-supported [C60]-fullerenes an alternative to Ru(ii) polypyridyls for photodynamic solar water disinfection? Photochem. Photobiol. Sci. 13, 397–406.

Misba, L., Kulshrestha, S., Khan, A.U., 2016. Antibiofilm action of a toluidine blue O-silver nanoparticle conjugate on Streptococcus mutans: a mechanism of type I photodynamic therapy. Biofouling 32, 313–328.

Nafee, N., Youssef, A., El-Gowelli, H., Asem, H., Kandil, S., 2013. Antibiotic-free nanotherapeutics: Hypericin nanoparticles thereof for improved in vitro and in vivo antimicrobial photodynamic therapy and wound healing. Int. J. Pharm. 454, 249–258.

Perni, S., Prokopovich, P., Parkin, I.P., Wilson, M., Pratten, J., 2010. Prevention of biofilm accumulation on a light-activated antimicrobial catheter material. J. Mater. Chem. 20, 8668–8673.

Redmond, R.W., Gamlin, J.N., 1999. A compilation of singlet oxygen yields from biologically relevant molecules. Photochem. Photobiol. 70, 391–475.

Sherwani, M.A., Tufail, S., Khan, A.A., Owais, M., 2015. Gold nanoparticle-photosensitizer conjugate based photodynamic inactivation of biofilm producing cells: Potential for treatment of C. albicans infection in BALB/c mice. PLoS One 10, 1–20.

Tawfik, A.A., Alsharnoubi, J., Morsy, M., 2015. Photodynamic antibacterial enhanced effect of methylene blue-gold nanoparticles conjugate on Staphylococcal aureus isolated from impetigo lesions in vitro study. Photodiagnosis Photodyn. Ther. 12, 215–220.

Vilsinski, B.H., Gerola, A.P., Enumo, J.A., Campanholi, K.D.S.S., Pereira, P.C.D.S., Braga, G., Hioka, N., Kimura, E., Tessaro, A.L., Caetano, W., 2015. Formulation of aluminum chloride phthalocyanine in Pluronic^TM^ P-123 and F-127 block copolymer micelles: Photophysical properties and photodynamic inactivation of microorganisms. Photochem. Photobiol. 91, 518–525.

Vimaladevi, M., Divya, K.C., Girigoswami, A., 2016. Liposomal nanoformulations of rhodamine for targeted photodynamic inactivation of multidrug resistant gram negative bacteria in sewage treatment plant. J. Photochem. Photobiol. B Biol. 162, 146–152.

Wilkinson, F., Helman, W.P., Ross, A.B., 1993. Quantum Yields for the Photosensitized Formation of the Lowest Electronically Excited Singlet State of Molecular Oxygen in Solution. J. Phys. Chem. Ref. Data 22, 113–262.

Yao, T., Wang, J., Xue, Y., Yu, W., Gao, Q., Ferreira, L., Ren, K.-F., Ji, J., 2019. A photodynamic antibacterial spray-coating based on the host–guest immobilization of the photosensitizer methylene blue. J. Mater. Chem. B 7, 5089–5095.

1. p= reduction on CFU counts from **planktonic** cultures.

   ba= prevention of **biofilm adhesion**/growth.

   mb= reduction on CFU counts after treatment of mature biofilm.

   n.s.: not specified. [↑](#footnote-ref-1)
2. 1 The values informed here correspond to the higher quantum yields of singlet oxygen Φ_∆_ achieved by the SPS measured in aqueous media unless otherwise noticed. [↑](#footnote-ref-2)
